# Autologous Fat Transfer as a Treatment for Peripheral Neuropathic Pain without Apparent Cause

**DOI:** 10.1097/GOX.0000000000001905

**Published:** 2018-08-16

**Authors:** Jip Beugels, Juliette E. Hommes, Andrea J.R. Balthasar, René R.W.J. van der Hulst, Andrzej A. Piatkowski de Grzymala

**Affiliations:** From the * Department of Plastic, Reconstructive and Hand Surgery, Maastricht University Medical Center+, Maastricht, The Netherlands; † NUTRIM School of Nutrition and Translational Research in Metabolism, Maastricht University, Maastricht, The Netherlands; ‡ Department of Anesthesiology and Pain Medicine, Maastricht University Medical Center+, Maastricht, The Netherlands; § MHeNs School for Mental Health and Neuroscience, Maastricht University, Maastricht, The Netherlands.

## Abstract

Neuropathic pain has a far-reaching effect on the daily lives of patients. Recently, autologous fat transfer (AFT) has demonstrated promising results in patients with painful scars or after neuroma excision. However, there is a subgroup of patients who do not show any apparent cause for the pain. We hypothesized that in these patients, AFT alone in the area around the affected nerve might lead to beneficial results. Patients with clearly demarcated neuropathic pain and who had exhausted all other treatment options were referred by a pain specialist. Fourteen patients who met the inclusion criteria received AFT in the area of the affected nerve. Pain scored on the visual analog scale, patient satisfaction, and quality of sleep were recorded before and after surgery. To investigate long-term effects, a second follow-up was planned at least 1 year later. Patient satisfaction was 93% after the first follow-up and 86% after more than 1 year. The mean VAS score was 7.4 before surgery and significantly decreased to 3.8 after autologous fat grafting (*P* < 0.0001) and 4.3 (*P* = 0.0017) at long-term follow-up. The quality of sleep improved in 50% of the patients, whereas the remainder indicated no difference. No complications were registered. The results show that AFT alone, even over a longer period of time and in patients refractory to multiple treatment modalities, can be useful to treat peripheral neuropathic pain without apparent cause. For definitive evidence, a larger prospective study is warranted.

## INTRODUCTION

Neuropathic pain, estimated to occur in 1–8% of the general population, is a challenge in daily practice with a profound influence on daily life.^1^ Treatment modalities are diverse and include various forms of pharmacotherapy, electrical stimulation, and surgical interventions.^2–4^ Pharmacotherapy and other nonsurgical treatments lead to a clinically meaningful pain reduction in less than 50% and 30% of the cases, respectively.^5,6^ Patients with refractory pain often undergo surgery, which traditionally was mostly performed by resection and relocation of the affected nerve into muscle, vein, or bone.^7–9^ In recent years, autologous fat transfer (AFT) has been introduced into the arsenal of surgical techniques to treat neuropathic pain caused by neuromas, burns and scars, and postmastectomy pain syndrome.^10–16^ Acknowledging the beneficial effect of AFT found for these indications, our aim was to investigate whether AFT can be an effective treatment for peripheral neuropathic pain when there is no clear cause in the form of a neuroma or scar.

## METHODS

A retrospective cohort study was conducted among patients with clearly demarcated nonneuroma neuropathic pain who were treated with AFT between December 2011 and August 2014.

Institutional review board approval was obtained under number METC 16-04-015.

### Patient Inclusion

All patients were referred by a pain specialist and were diagnosed with neuropathic pain that was refractory to multiple treatment modalities. The majority of the patients presented with a history of trauma or a surgical procedure in the area before the onset of the pain. Preoperative examination included Tinel’s sign and completion of the visual analog scale (VAS) as an assessment of pain intensity. Inclusion criteria were a clearly demarcated area of pain and positive Tinel’s sign in the absence of obvious scars or neuromas as validated by means of high-resolution ultrasonography.^17^

### Surgical Procedure

Surgery was performed under general anesthesia. The donor site (usually abdomen) was infiltrated with saline (NaCl 0.9%) and 0.001 mg adrenaline/ml before adipose tissue was harvested through a 3-mm blunt Mercedes tip cannula, at low suction settings using a Cytori pure graft system (Cytori Therapeutics Inc., San Diego, Calif.). The lipoaspirate was then reinjected subcutaneously in the preoperatively marked area using a fanning technique.

### Outcome Measures

Baseline characteristics were recorded. Primary outcome measures were patient satisfaction (yes or no) and pain scores pre- and postoperatively on the VAS ranging from 0 “no pain at all,” to 10 “unbearable pain.” A long-term follow-up was planned after a minimum of 1 year after surgery to evaluate changes in pain experience. Quality of sleep and safety of the procedure were also evaluated.

### Statistical Analysis

Statistical comparison of the pre- and postoperative VAS scores was performed by paired Student’s *t* test. A *P* value < 0.05 was considered to be statistically significant.

## RESULTS

**Fig. 1.**
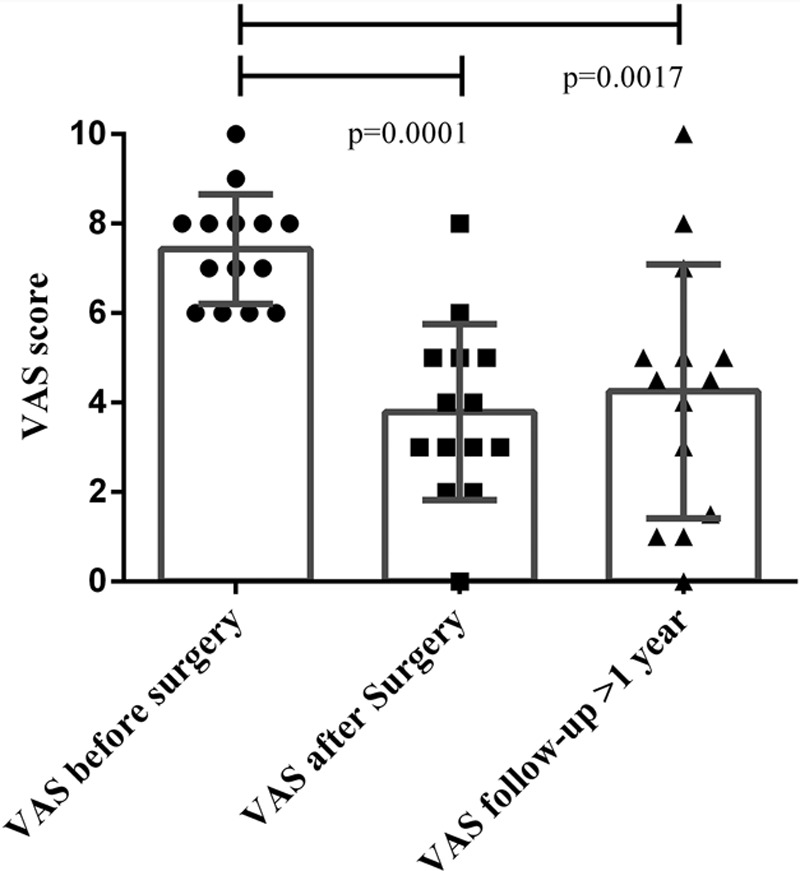
VAS score before surgery and at the 2 follow-ups. The mean VAS score significantly decreased from 7.4 (range, 6–10) before surgery to 3.8 (range, 0–8) after autologous fat grafting at 8-week follow-up and 4.3 (range, 0–10) at the follow-up of more than 1 year. The VAS increased in 3 cases between the first and second follow-up but was still below the preoperative value.

Fourteen individual patients were included. Their baseline characteristics can be found in Table 1. Thirteen patients (93%) were satisfied with the treatment results at the first postoperative follow-up, whereas 11 patients (86%) remained satisfied after more than 1 year follow-up. The mean VAS score was 7.4 before surgery (range, 6–10) and significantly decreased to 3.8 after autologous fat grafting (*P* < 0.0001; range, 0–8; Fig. 1) at 8-week follow-up and 4.3 (*P* = 0.0017; range, 0–10) at long-term follow-up (on average 28 mo). In 3 patients, the VAS increased between the first and second follow-up but was still below the preoperative value. The quality of sleep improved in 50% of the patients, whereas the remainder indicated no difference. No complications were registered.

**Table 1.**
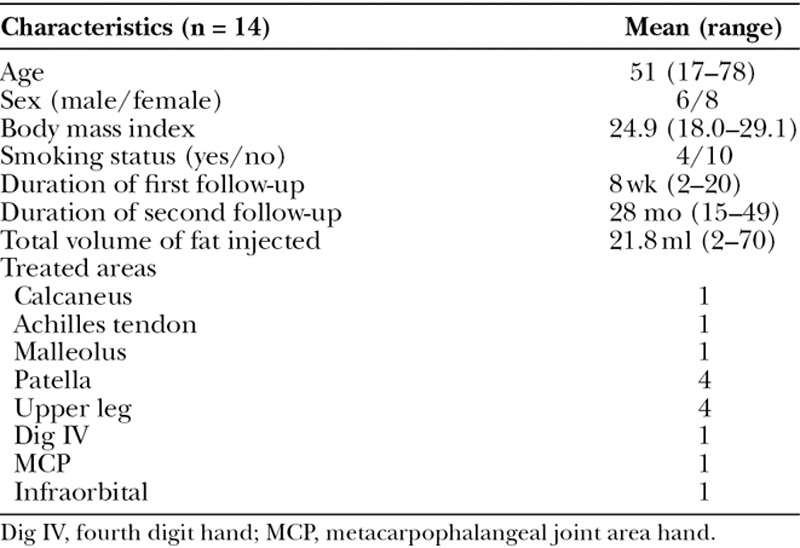
Patient Demographics

## DISCUSSION

The study results revealed that the vast majority of patients (86%) were satisfied with the pain relief after more than 1 year follow-up and 50% of the patients reported improved quality of sleep. In the literature, a pain reduction of at least 30% is generally regarded as being clinically relevant.^3^ This effect was reached in 9/14 patients (64%), whereas 5/14 patients (36%) reported a pain reduction of at least 50%. The meaningful effect of the procedure was corroborated by the fact that previous “standard care” pain treatments had either no or short-lasting effects, whereas the effect of AFT lasted much longer.

The significant decrease in pain reflects the results from studies wherein cause of the pain was known. Vaienti et al.^10^ reported a reduction of 23.2% in the mean disabilities of the arm, shoulder, and hand scores and an improvement in the VAS score of 22% (although not statistically significant) after perineural fat grafting in 8 patients for the treatment of painful end neuromas of the upper limb. Fredman et al.^14^ noted a pain reduction in 6 of 7 patients with burn scars. Huang et al.^16^ reported a significant decrease in VAS score of 4.38 after 1 week, 5.38 after 4 weeks, and 5.62 after 24 weeks of treatment, and a likewise decrease in the Neuropathic Pain Symptom Inventory in 13 patients with painful scars. In patients with postmastectomy pain syndrome, several studies revealed that AFT significantly decreased the VAS score versus nonoperated controls, with a mean decrease of >3 points.^11,12,18^

Interestingly, all 14 patients reported pain relief in the first weeks after surgery. However, 2 patients indicated recurrence of pain after 2 months with an increased VAS score compared with preoperative. Another patient reported at the long-term follow-up that the VAS had been rising slightly after a few months but was still at acceptable levels at the last follow-up. Although Huang et al.^16^ found no recurrence of pain in patients responding to treatment, Vaienti et al.^10^ described a similar deterioration of the pain relief over time in some patients. We hypothesize that graft volume loss, in literature described to be between 40% and 80% in the first months postoperative, may be responsible for the recurrence of pain.^19–21^ If that is the case, treatments or methods, which improve graft volume retention, may also improve the long-term effects on pain.

## CONCLUSIONS

This study shows that AFT is a useful alternative in localized peripheral neuropathic pain with a high patient-reported satisfaction even after 1 year of follow-up. Nevertheless, prospective studies with larger study populations are required to provide definitive evidence.

## ACKNOWLEDGMENTS

We would like to thank the Department of Anesthesiology of Maastricht University Medical Center and especially Dr. Balthasar and Dr. Sommer.
